# Supplementary material for: Multi-Omics Reveals the Role of Arachidonic Acid Metabolism in the Gut–Follicle Axis for the Antral Follicular Development of Holstein Cows
Source: Int J Mol Sci. 2024 Sep 1;25(17):9521. doi: 10.3390/ijms25179521 (PMC11395146; doi:10.3390/ijms25179521)
Supplement: Supplementary file 1 [file ijms-25-09521-s001.zip › ijms-3117833-supplementary.pdf]

Multi-omics reveals the role of arachidonic acid metabolism in the gut–follicle axis for the antral follicular development of Holstein cows

Yajun Guo, Shiwei Wang, Xuan Wu, Rong Zhao, Siyu Chang, Chen Ma, Shuang Song, Shenming Zeng*
State Key Laboratory of Animal Biotech Breeding, National Engineering Laboratory for Animal Breeding, Key Laboratory of Animal Genetics, Breeding and Reproduction of the Ministry of Agriculture, College of Animal Science and Technology, China Agricultural University, Beijing, 100193, China

Materials and Methods

Using a vaginal vault ultrasound puncture device for follicular

The protocol for transvaginal ultrasound-guided follicle aspiration and the method for determining follicle size were collected concerning. To ensure consistency in follicle size, the probe attached is used a vagina to visualize ovarian follicles for transvaginal aspiration. The veterinary ultrasound machine's measuring imaging system (Easi-Scan Micro-Convex, BCF Technology Ltd, Scotland, UK; Probe parameter: 15 mm radius, frequency range 5 MHz to 8 MHz, 80 element crystal array, 10 digital channels, 90º curved) guided the aspiration of follicular fluid from the follicles that could be satisfied with follicles (Diameter: 4-8mm) were collected from each cow. To be specific, transvaginal aspiration is performed with a transvaginal probe handle into which the ultrasound probe is mounted. The probe handles had a channel for the needle. In the tranquilized cow, it is placed in the vagina, and the ovary is manipulated via palpation per rectum. A specialized needle (18 gauge needle) is placed through the guide channel of the probe handle. The ovary is imaged through the vaginal wall and manipulated by the hand per rectum so that the follicle is placed in the path of the needle as visualized on the ultrasound screen. The needle is then guided forward through the vaginal wall into the follicle, and the contents of the follicle are aspirated, typically with a vacuum pump.

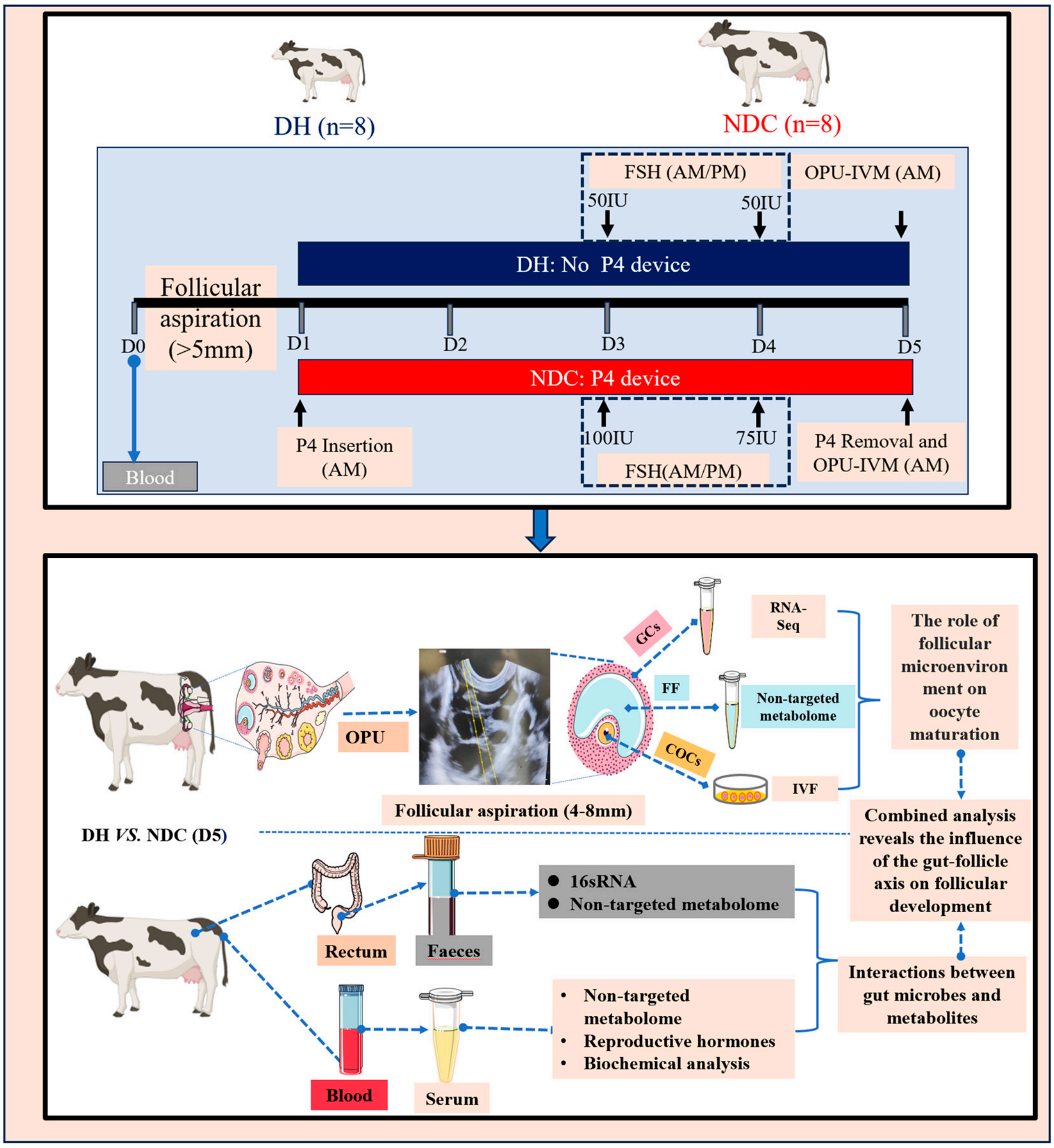

Figure S1 Experimental design and protocols. DH, dairy heifer; NDC, non-lactating multiparous dairy cows; OPU, ovum pick up; FSH, follicle-stimulating hormone; P4 device, slow-releasing progesterone device; GCs, granulosa cells. FF, follicular fluid; IVF, *in vitro* fertilization.

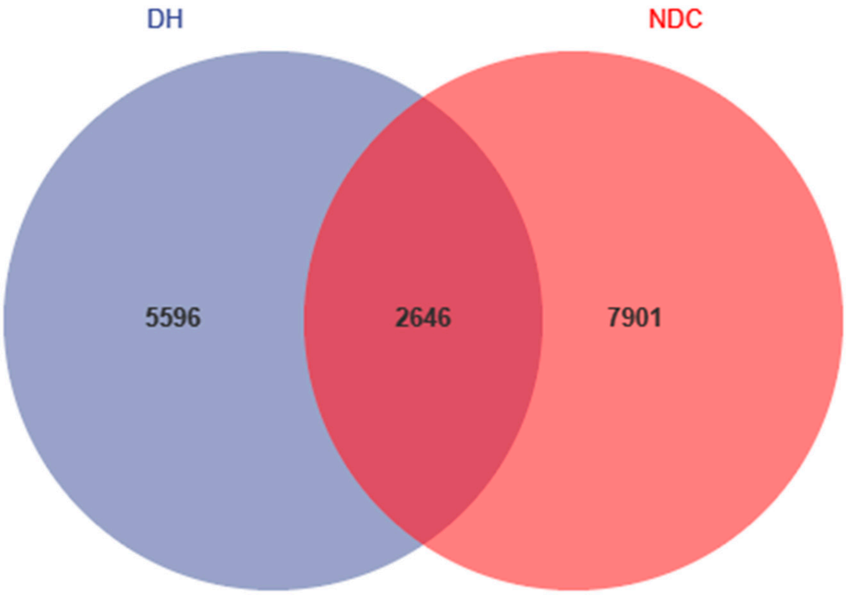

Figure S2 The Venn diagram of microbiota between DH and NDC groups. DH, dairy heifer; NDC, non-lactating multiparous dairy cows.

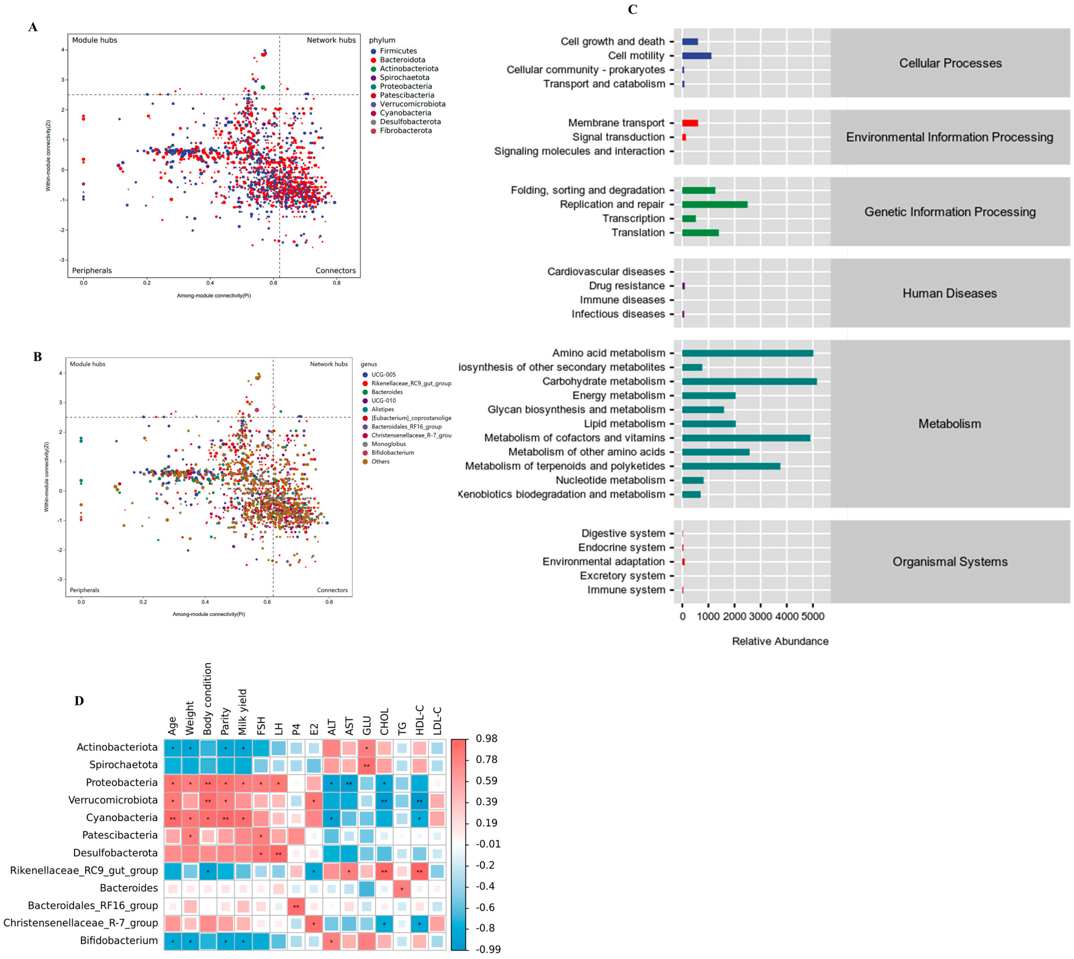

Figure S3 The different gut microbes, their potential function, and correlations with reproductive hormones and key biochemicals. **A** The ZiPi diagram analyzes differential gut microbes in the phylum level between DH and NDC groups. **B** The ZiPi diagram analyzes differential gut microbes at the genus level between DH and NDC groups. The node size is proportional to its abundance (in $\log_2$ (CPM/n), and nodes belonging to the top 5 classification units with the highest abundance are identified by different colors. **C** Prediction of the potential function of differential gut microbes. **D** Correlation of microbes with reproductive hormones and key biochemical. **P* < 0.05, ***P* < 0.01.

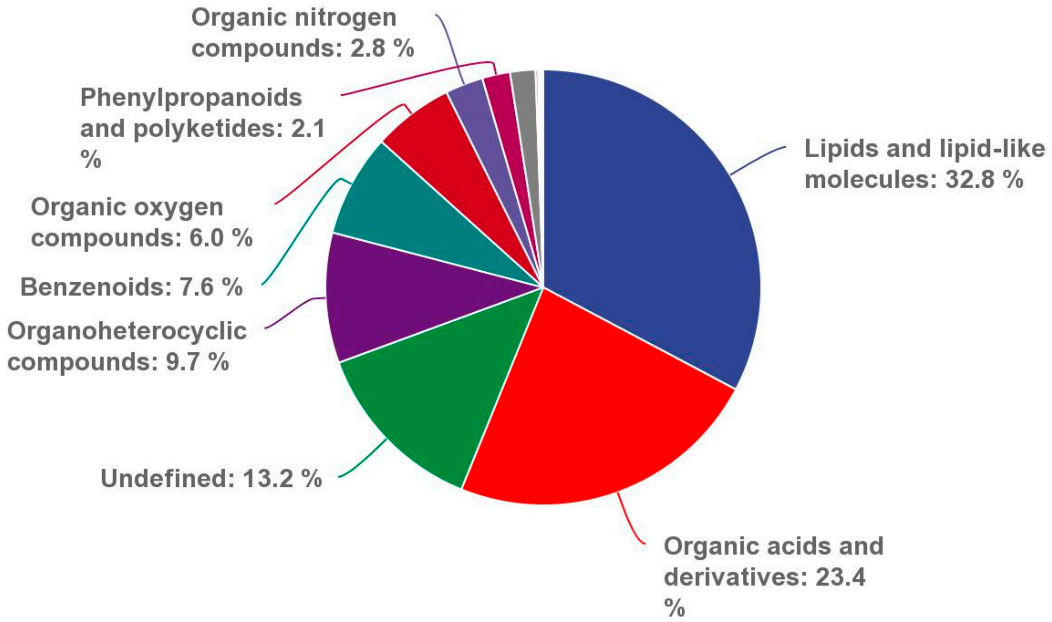

Figure S4 Classification and proportion of serum metabolites between DH and NDC groups.

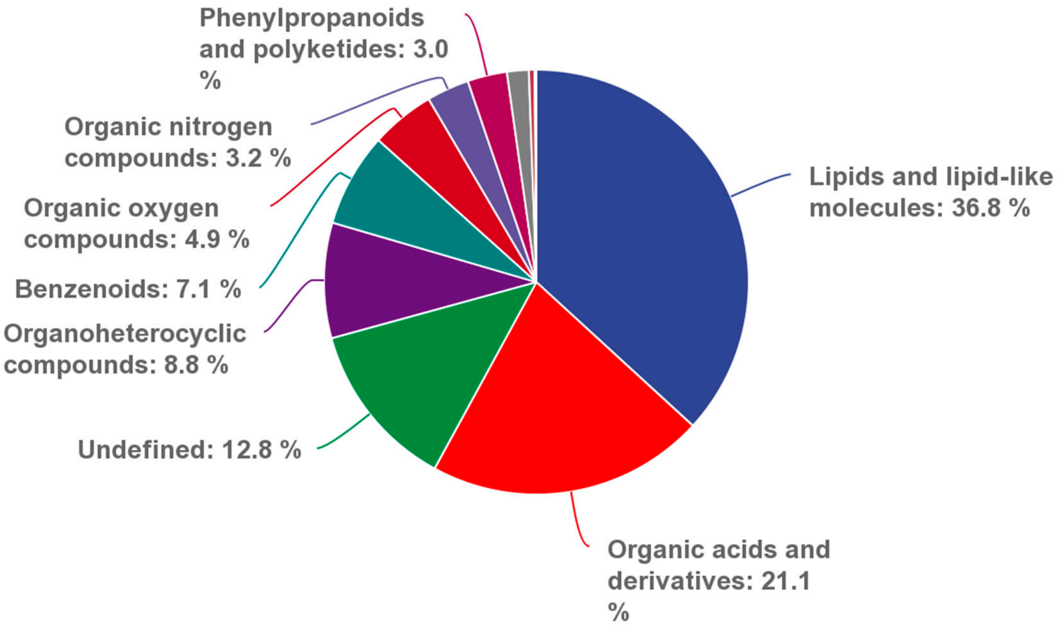

Figure S5 Classification and proportion of serum metabolites between DH and NDC groups.

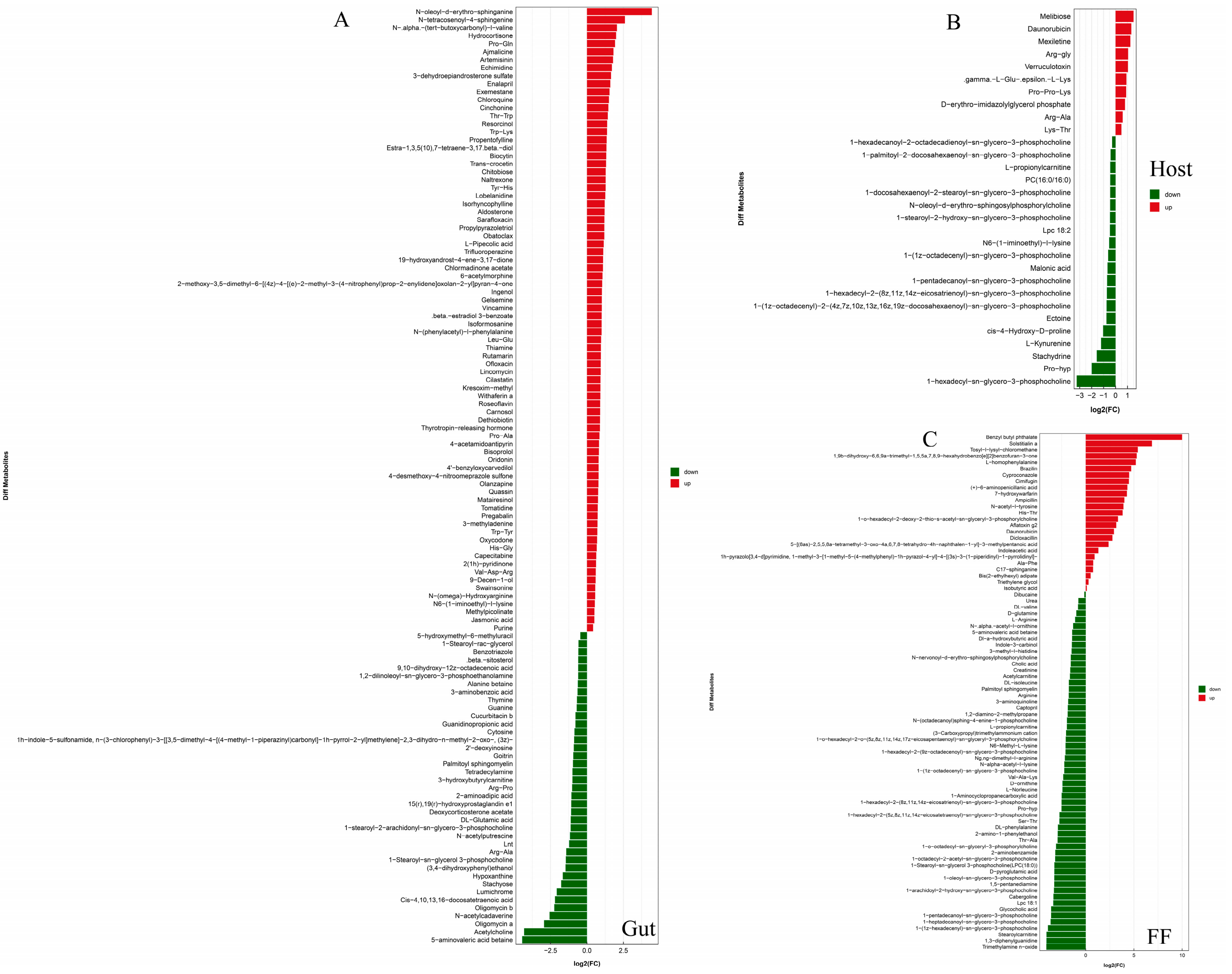

Figure S6 Metabolic profiles of gut microbe-related metabolites, serum metabolites, and follicular fluid metabolites in the DH and NDC groups. **A** Bar graphs show the gut microbial differential metabolites in the NDC group compared to the DH group. **B** Bar graph showing differential metabolites in serum for the NDC group compared to the DH group. **C** Bar graph showing differential metabolites in follicular fluid comparing in the NDC group compared to the DH group.

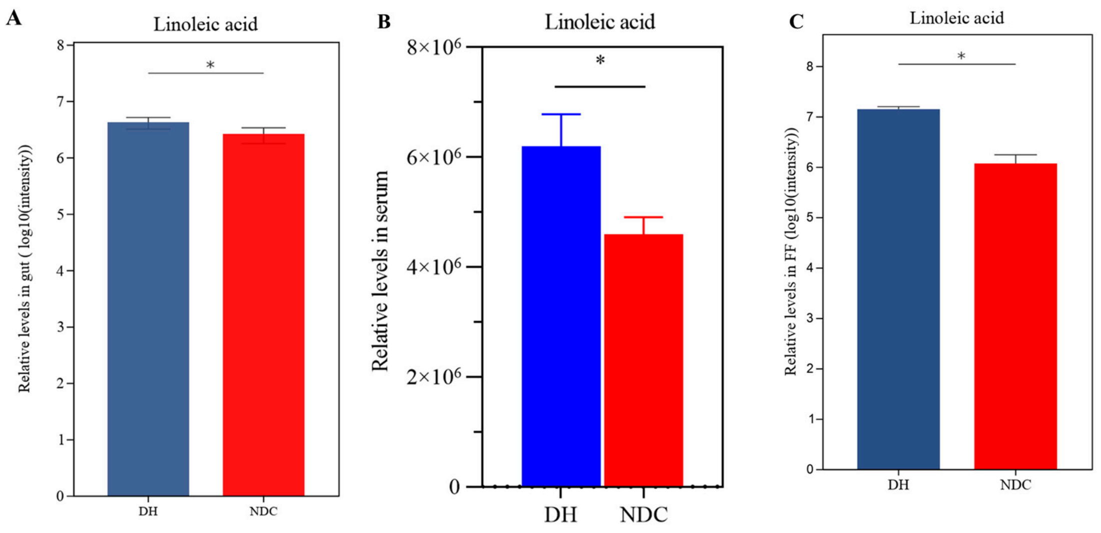

Figure S7 Linoleic acid levels of the gut, serum, and follicular fluid in the DH and NDC groups. **A** Linoleic acid levels of the gut between DH and NDC groups. **B** Linoleic acid levels of serum in the DH and NDC groups. **C** Linoleic acid levels of Follicular fluid metabolites (FF) for the DH and NDC groups.

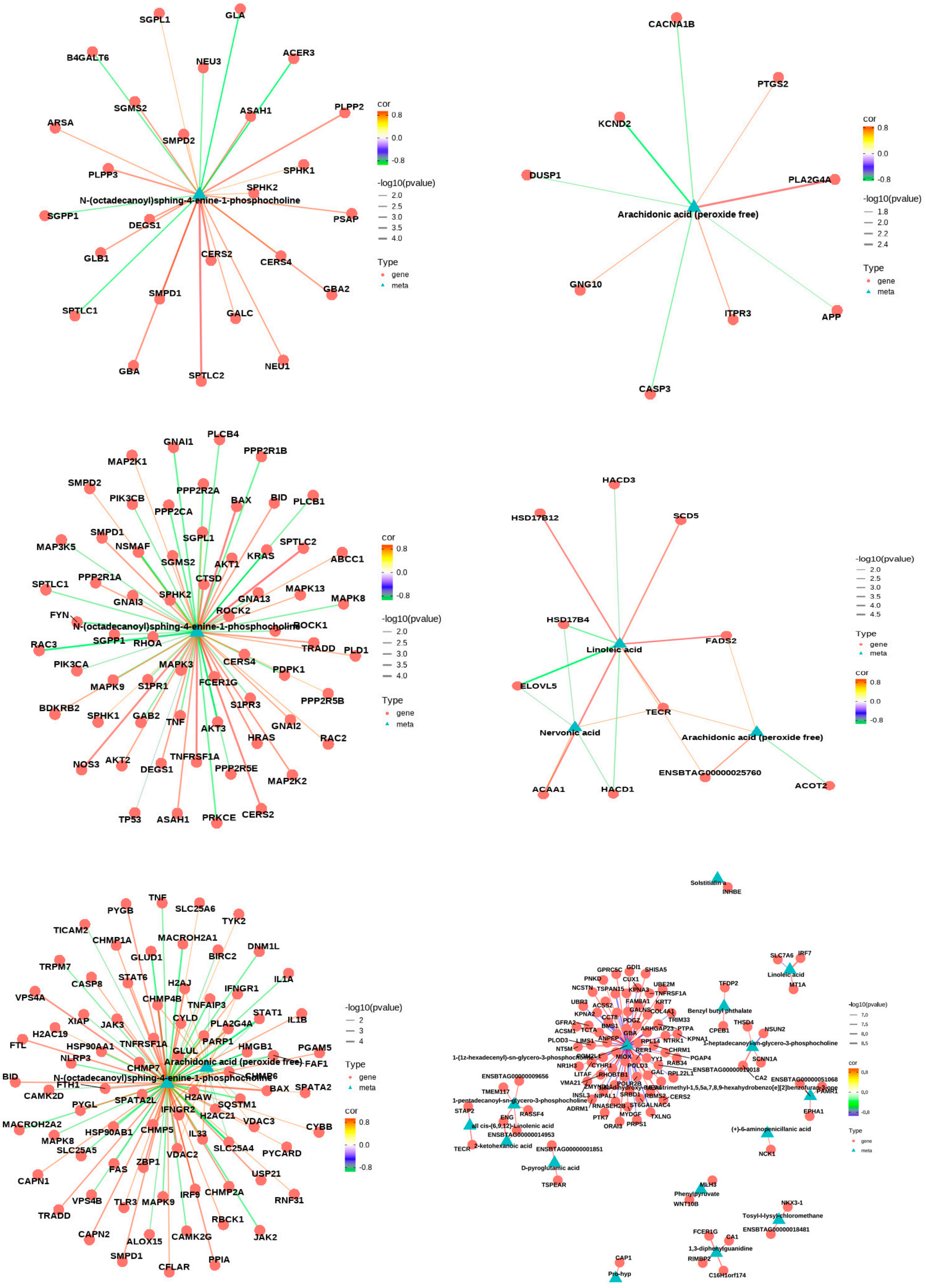

Figure S8 Network diagram of arachidonic acid, linoleic acid, and phosphocholine metabolites interactions with genes. The correlation coefficients were calculated by Spearman's correlation coefficient(|R|>0.8, P < 0.05). The red line indicates a positive correlation; the green line indicates a negative correlation. The line thickness represents the P-value size.

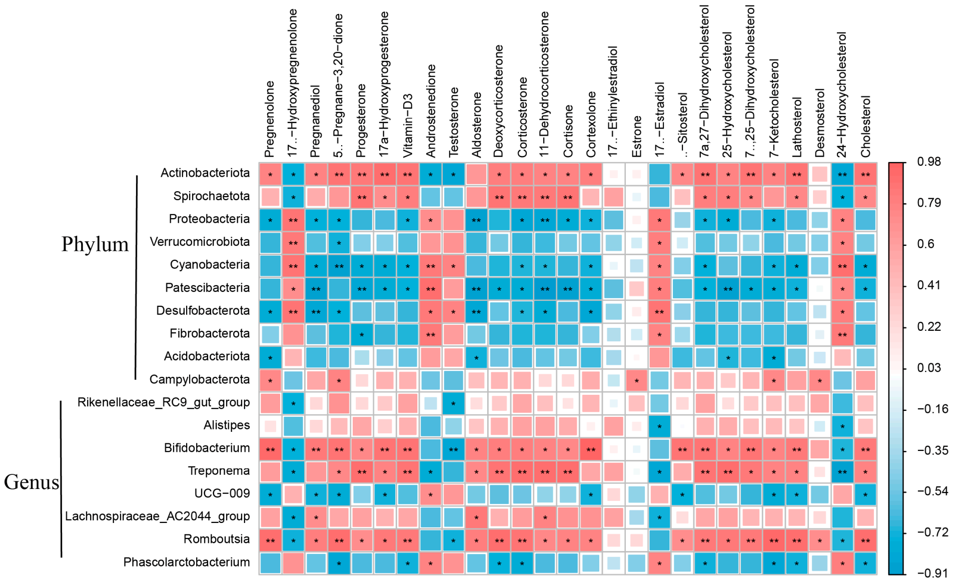

Figure S9 Correlation analysis of gut microbiota with the steroid hormones of follicular fuid. The red indicates a positive correlation and the blue indicates a negative correlation. *P < 0.05, **P < 0.01.

Negative control

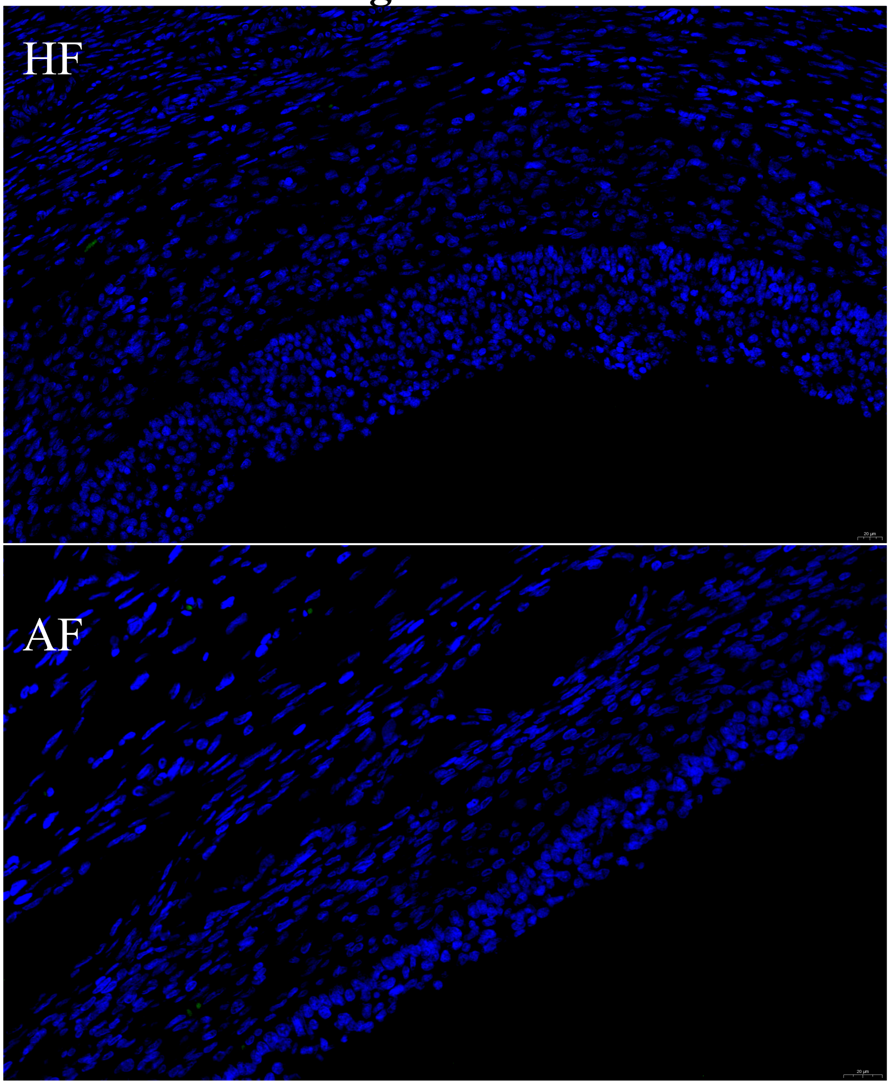

Immunofluorescence negative control image

Table S1. The basic information and serum concentrations of reproductive hormones in experimental dairy cattle

Items	DH (n=8)	NDC (n=8)
Age (Y)	1.14 ± 0.75	5.75 ± 1.98
Weight (Kg)	349.66 ± 26.36	707.50 ± 30.72
Body condition	3.23 ± 0.02	3.07 ± 0.28
Parity	NA	3.38 ± 1.80
Milk yield (Kg)	NA	17423.00 ± 1788.63
FSH (mIU/ml)	1.34 ± 0.43	1.58 ± 0.28
LH (mIU/ml)	5.31 ± 0.96	11.87 ± 3.92
P_4 (ng/ml)	6.08 ± 1.92	13.15 ± 2.82
E_2 (pg/ml)	60.49 ± 5.67	76.24 ± 4.66

Table S2. Primer sequences of different genes for qPCR

Genes	primer sequences	NCBI accession No.
CYP19A1	F: GCTTTTGGAAGTGCTGAACCCAAGG<br>R: GGGCCCAATTCCCAGAAAGTAGCTG	NM_174305.1
Cyp11A1	F: TGTCTCAGGACTTCGTCAGC<br>R: ACAGTTCTGGAGGGACGTTG	NM_176644.2
CYP17A1	F: GCATTCCTGACGTCAATGACAGAG<br>R: TGTTGGTGGAGGAGAAGGATCTG	NM_174304.3
HSD17B2	F: GCTGAACATCACCAACACGC<br>R: ACAGCCCCGAAGAAGTTCAC	NM_001075726.1
HSD3B1	F: TCCGGGTGCTAGACAAAGTC<br>R: CGGTGTGGATGACCACTGAG	NM_174343.3
PTGS2	F: CCCACTTCAAAGGAGTCTGG<br>R: TGGTGGACTCTCAATCAAATG	NM_174445.2
PTGES	F: ATGGCCCTTTGAGATTGTGA<br>R: CTGTTTGCTTTTCCCAGCAT	NM_174443.2
ALOX5	F: ACCATCGAGCAGATTGTGGAA<br>R: GGCTCAGGTGGCTTCTCAGAT	NM_001192792.2
PLCB1	F: CACAAAGAAATCCGCCAGCAG<br>R: GGGGCAGAGCTGTGATTGCT	NM_174817.1
MIF	F: GCAGAACCGCTCCTACAG<br>R: CGTTCATGTCGCAGAAGTTG	NM_001033608.1
CASP3	F: TACTTGGGAAGGTGTGAGAAAACTAA<br>R: AACCCGTCTCCCTTTATATTGCT	NM_001077840.1
IL6	F: GCTCTCATTAAGCGCATGGT<br>R: CCTTGCTGCTTTCACACTCA	NM_173923.2
GAPDH	F: GGGTCATCATCTCTGCACCT<br>R: GGTCATAAGTCCCTCCACGA	NM-001034034.2

Table S3. Antibodies lists used in this paper

Primary antibodies	Vendor	Dilution	Source
PTGS2/COX2 (WB/IF)	Proteintech (12375-1-AP)	1:500/1:200	Rabbit
ALOX5 (WB/IF)	Proteintech (10021-1-Ig)	1:500/1:100	Rabbit
PLCB1 (WB/IF)	Proteintech (26551-1-AP)	1:500/1:200	Rabbit
GAPDH (WB)	Affinity (T0004)/Proteintech (60004-Ig)	1:1000	Rabbit/Mouse
SOD1 (WB)	Santa(sc-271014)	1:500	Mouse
HIF-1α (WB)	CST (36169)	1:1000	Rabbit
ERK1/2 (WB)	Absin (abs130092)	1:2000	Rabbit
P-ERK1/2 (WB)	Proteintech (28733-1-AP)	1:2000	Rabbit
P38MAPK (WB)	CST (8690)	1:1000	Rabbit
BCL2 (WB)	Abmart (T40056)	1:1000	Rabbit
BAX (WB)	Beyotime (AB026-1)	1:500	Rabbit
CASP3 (WB)	Proteintech (1967-1-AP)	1:1000	Rabbit
IRF1 (WB)	CST (8478)	1:1000	Rabbit
RIPK1 (WB)	Affinity (DF10141)	1:1000	Rabbit
GSDMD (WB)	Affinity (AF4012)	1:1000	Rabbit
IL-1B (WB)	Invitrogen (ASC0912)	1:1000	Rabbit
P53 (WB)	CST (2527)	1:1000	Rabbit
MIF (WB)	Proteintech (20415-1-AP)	1:500	Rabbit
**Secondary antibodies**			
HRP-conjugated goat anti-rabbit IgG (WB)	CST (7074S)	1:2000	Goat
HRP-conjugated horse anti-mouse IgG (WB)	CST (7076S)	1:2000	Horse
Fluor 488 conjugated goat anti-rabbit IgG (IF)	Invitrogen (A-11008)	1:200	Goat

Table S4. Standard curve of steroidogenesis-related metabolites

Metabolite Name	Retention Time (min)	Equation	**R**
11-Hydroxyandrosterone	3.45	y = 0.01905 x + 1.54298	0.99620
11-Ketoetiocholanolone	3.59	y = 0.02200 x + 0.00283	0.99504
Testosterone	4.18	y = 0.01350 x + 0.00111	0.99922
Androstenedione	4.42	y = 0.01215 x + 2.79610	0.99893
dehydroepiandrosterone	4.63	y = 0.00807 x + 0.01334	0.99641
Dihydrotestosterone	5.11	y = 0.00216 x + 5.64021	0.99874
Androstanedione	5.29	y = 0.00406 x + 6.75876	0.99592
5β-Androsterone	5.53	y = 0.00891 x + 0.00934	0.99224
Androsterone	5.68	y = 0.01495 x + 0.00332	0.99502
Aldosterone	1.33	y = 5.26481e-4 x + 1.11085	0.99260
Cortisol	1.66	y = 9.13398e-4 x + 5.14872	0.99337
Cortisone	1.69	y = 0.01062 x + 4.57429	0.99680
11-Dehydrocorticosterone	2.47	y = 0.00640 x + 4.45571	0.99921
Corticosterone	2.84	y = 0.00509 x + 4.28450	0.99617
Cortexolone	3.05	y = 0.00103 x - 1.9649	0.99910
Deoxycorticosterone	4.28	y = 0.01565 x + 9.50931	0.99875
16α-Hydroxy-Estrone	2.08	y = 0.00225 x - 1.56110	0.99908
2-Hydroxy-Estrone	3.33	y = 0.00343 x - 1.47783	0.99899
17β-Estradiol	3.99	y = 0.00782 x + 0.00923	0.99045
17a-Estradiol	4.27	y = 0.01314 x + 0.00836	0.99080
17α-Ethinylestradiol	4.38	y = 1.73392e-4 x + 1.25169	0.99467
Estrone	4.43	y = 0.01081 x + 0.00681	0.99292
4-Methoxy-Estrone	4.54	y = 0.00491 x + 4.06797	0.99673
2-Methoxy-Estrone	4.66	y = 0.00322 x + 0.00149	0.99373
17α-Hydroxypregnenolone	4.52	y = 0.00965 x + 0.00264	0.99612
17a-Hydroxyprogesterone	4.64	y = 0.00570 x + 1.13918	0.99721
Progesterone	5.91	y = 0.01313 x + 7.21921	0.99882
Pregnenolone	6.14	y = 0.00713 x + 0.00325	0.99382
Pregnanediol	6.50	y = 2.89965e-4 x + 1.39795	0.99628
5α-Pregnane-3,20-dione	6.69	y = 7.73784e-4 x + 1.05518	0.99433
7-Hydroxy-cholesten-3-one	10.01	y = 0.00856 x + 0.00272	0.99465
7-Ketocholesterol	10.14	y = 0.00534 x + 0.00145	0.99879
Desmosterol	11.10	y = 0.00161 x + 0.26104	0.99232
Lathosterol	11.66	y = 0.00143 x + 0.04184	0.99546
Cholesterol	11.67	y = 0.00159 x + 0.03643	0.99536
β-Sitosterol	12.17	y = 9.55465e-4 x - 0.00147	0.99727
24,25-Dihydrolanosterol	12.44	y = 9.79438e-4 x - 0.00170	0.99928
7α,25-Dihydroxycholesterol	6.97	y = 4.82395e-4 x + 0.00402	0.99711
7a,27-Dihydroxycholesterol	7.28	y = 0.00908 x + 0.00303	0.99254
25-Hydroxycholesterol	8.90	y = 0.00352 x + 0.00983	0.99664
24-Hydroxycholesterol	9.02	y = 0.00395 x + 0.00359	0.99570
20α-Hydroxycholesterol	9.32	y = 0.00902 x + 0.03298	0.99983
Vitamin-D3	11.20	y = 1.86893e-4 x - 5.51956	0.99557

Table S5. Standard curve of arachidonic acid-related metabolites

Metabolite Name	Mass Info	Retention Time (min)	Equation	R
Arachidonic acid	303.2 / 259.2	7.381651664	y = 0.03562 x + 0.17307	r = 0.99752
Docosahexaenoic acid	327.1 / 229.2	7.208752169	y = 0.00617 x + -0.19746	r = 0.99780
12(S)-HETE	319.1 / 179.0	5.452912962	y = 0.01474 x + -0.18418	r = 0.99962
15(S)-HETE	319.1 / 219.0	5.205975636	y = 0.00256 x + -0.04808	r = 0.99907
14(15)-EpETE	317.1 / 207.0		y = 0.00142 x + 0.01062	r = 0.99887
9(S)-HODE	295.1 / 171.1	5.109136402	y = 0.00746 x + -0.00334	r = 0.99861
13(S)-HODE	295.0 / 195.0	5.065040957	y = 0.01122 x + 3.17056e-4	r = 0.99885
Leukotriene B4	335.2 / 195.0	3.992798151	y = 0.01153 x + -0.08347	r = 0.99974
Leukotriene D4	495.3 / 177.2		y = 0.00879 x + 0.16994	r = 0.99899
6-keto-Prostaglandin F1α	369.3 / 163.0	0.835489053	y = 0.01976 x + -0.06212	r = 0.99960
8-iso-Prostaglandin F2α	353.1 / 309.3	1.971293193	y = 0.03177 x + -0.05297	r = 0.99865
Prostaglandin D2	351.2 / 271.3	2.154006367	y = 0.02592 x + -0.62552	r = 0.99853
Prostaglandin E2	351.2 / 271.3	2.525591049	y = 0.02337 x + -0.37165	r = 0.99830
Prostaglandin F2α	353.1 / 309.3	1.975457392	y = 0.02884 x + -0.50634	r = 0.99847
Thromboxane B2	369.2 / 169.0	1.583907984	y = 0.01273 x + -0.27681	r = 0.99739

Table S6. The difference in follicular numbers, OPU results, and early embryo development between DH and NDC groups

OPU	DH (n=8)	NDC (n=8)	*P*-value
The number of the follicle (4-8 mm) per cow	30.25 ± 9.70	21.13 ± 5.93	< 0.05
The numbers of OPU per cow	5	6	NA
The number of COCs per OPU per cow	20.08 ± 9.14	12.13 ± 7.47	< 0.05
The number of oocytes available for IVM per OPU per cow	16.25 ± 7.13	9.63 ± 5.33	< 0.05
Cleavage rates (%)	72.12 ± 0.07	41.45 ± 0.25	< 0.05
Blastocyst rates (%)	28.36 ± 4.43	16.10 ± 6.59	< 0.05

Table S7. The differential metabolites of gut microbiota between DH and NDC groups

Name	VIP (>4)	Fold change (NDC *VS* DH)	*P*-value	Class
Hypoxanthine	16.30	0.32	0.0005	Imidazopyridines
Linoleic acid	13.21	0.62	0.010905	Fatty Acyls
2-aminoadipic acid	11.36	0.48	0.008794	Carboxylic acids and derivatives
Lithocholic acid	9.08	2.00	0.005667	Steroids and steroid derivatives
Acetylcholine	7.62	0.05	0.007484	Organonitrogen compounds
His-ser	7.47	0.64	0.003382	Pyrimidine nucleosides
Rac-2-despiperidyl-2-aminorepaglinide	7.01	0.54	0.048129	Benzene and substituted derivatives
5-aminovaleric acid betaine	6.96	0.05	0.007394	Fatty Acyls
3-(3-Hydroxyphenyl) propanoic acid	6.89	0.21	0.00038	Phenyl propanoic acids
Methyl hexadecanoate	6.66	0.45	0.000518	Fatty Acyls
Uracil	6.15	0.47	0.000175	Diazines
21-Hydroxypregnenolone	6.05	0.14	0.000238	Steroids and steroid derivatives
Palmitic acid	5.94	0.89	0.001822	Fatty Acyls
Ile-Pro	5.89	0.57	0.001125	Pyrimidine nucleosides
11beta-hydroxyprogesterone	5.77	0.32	2.25E-05	Steroids and steroid derivatives
Pseudouridine	5.66	0.63	0.005861	Nucleoside and nucleotide analogues
Saccharin	4.88	0.62	0.008956	/
5.alpha. - androstan-3. alpha., 17beta.-diol-o-3-.beta.-glucuronic acid	4.87	0.49	0.000207	Steroids and steroid derivatives
Cinchonine	4.79	2.77	0.001067	/
Deoxyinosine	4.70	0.50	0.002523	Purine nucleosides
Lumichrome	4.62	0.24	0.000432	Pteridines and derivatives
Thymine	4.40	0.62	0.00061	Diazines
Desmethylverapamil	4.36	0.33	0.029206	Benzene and substituted derivatives
Hydrocortisone	4.23	4.00	0.000352	Steroids and steroid derivatives
Pro-Ala	4.16	1.81	0.00748	/
Artemisinin	4.12	3.46	0.001826	Prenol lipids

Table S8. The levels of serum metabolites between the DH and NDC groups

Name	VIP (>4)	Fold change (NDC VS. DH)	*P*-value	Class	Type
Cholesteryl sulfate	4.37	0.52	0.000359	Steroids and steroid derivatives	down
L-pyroglutamic acid	7.33	0.76	0.001046	Carboxylic acids and derivatives	down
D-allose	8.21	0.66	0.008526	Organooxygen compounds	down
Indoxyl sulfate	7.84	0.58	0.017366	Organic sulfuric acids and derivatives	down
Taurine	4.87	0.81	0.027133	Organic sulfonic acids and derivatives	down
L-dihydroorotate	6.12	0.69	0.028012	Carboxylic acids and derivatives	down
N-oleoyl-d-erythro-sphingosylphosphorylcholine	4.99	0.73	0.001058	Sphingolipids	down
1-hexadecyl-sn-glycero-3-phosphocholine	6.44	0.10	0.001489	Glycerophospholipids	down
Daunorubicin	5.59	2.50	0.003478		up
Melibiose	5.69	2.84	0.0043	Organooxygen compounds	up
Lpc 18:2	22.09	0.72	0.018498	Glycerophospholipids	down
1-pentadecanoyl-sn-glycero-3-phosphocholine	4.62	0.61	0.037113	Glycerophospholipids	down
1-hexadecanoyl-2-octadecadienoyl-sn-glycero-3-phosphocholine	11.38	0.82	0.039051	Glycerophospholipids	down

Table S9. The levels of FF metabolites between DH and NDC groups

Name	VIP (>4)	Fold change (NDC *VS.* DH)	*P*-value	Class	Type
Linoleic acid	25.52	0.09	0	Fatty Acyls	down
Benzyl butyl phthalate	14.24	1019.93	0	Benzene and substituted derivatives	up
Cimifugin	11.69	22.29	0	Benzopyrans	up
1-oleoyl-sn-glycero-3-phosphocholine	11.22	0.11	0.001	Glycerophospholipids	down
2-ketohexanoic acid	10.23	0.13	0	Keto acids and derivatives	down
C17-sphinganine	8.54	1.70	0.017	Organonitrogen compounds	up
Palmitoyl sphingomyelin	8.43	0.30	0.001	Sphingolipids	down
Lpc 18:1	7.10	0.10	0.002	Glycerophospholipids	down
Arginine	7.01	0.30	0.002	Carboxylic acids and derivatives	down
D-fructose	6.89	0.20	0	Organooxygen compounds	down
Curcumin	6.85	0.21	0	Diarylheptanoids	down
1,3-diphenylguanidine	6.48	0.06	0		down
Arachidonic acid (peroxide free)	6.12	0.38	0.001	Fatty Acyls	down
Creatinine	5.74	0.33	0	Carboxylic acids and derivatives	down
Solstitialin	5.31	117.01	0	Prenol lipids	up
all cis-(6,9,12)-Linolenic acid	5.13	0.15	0		down
Taurine	5.03	0.13	0.001	Organic sulfonic acids and derivatives	down
1-(1z-hexadecenyl)-sn-glycero-3-phosphocholine	4.98	0.07	0	Glycerophospholipids	down
Dl-lactate	4.90	0.02	0	Hydroxy acids and derivatives	down
Heptadecanoic acid	4.69	0.24	0.002	Fatty Acyls	down
N- (octadecanoyl)sphing-4-enine-1-phosphocholine	4.67	0.26	0	Sphingolipids	down
Ursocholic acid	4.63	0.37	0.022	Steroids and steroid derivatives	down
3-hydroxyphenylacetic acid	4.40	0.35	0	Phenols	down
1-o-hexadecyl-2-o-(5z,8z,11z,14z,17z-eicosapentaenoyl)-sn-glyceryl-3-phosphorylcholine	4.29	0.24	0.001	Glycerophospholipids	down
Hippuric acid	4.15	0.25	0.005	Benzene and substituted derivatives	down
Glycocholic acid	4.08	0.08	0.001	Steroids and steroid derivatives	down

L-Norleucine	4.04	0.19	0.003	Carboxylic acids and derivatives	down
